# Age Influences Loss Aversion Through Effects on Posterior Cingulate Cortical Thickness

**DOI:** 10.3389/fnins.2021.673106

**Published:** 2021-07-12

**Authors:** Zoe R. Guttman, Dara G. Ghahremani, Jean-Baptiste Pochon, Andy C. Dean, Edythe D. London

**Affiliations:** ^1^Department of Psychiatry and Biobehavioral Sciences, University of California, Los Angeles, Los Angeles, CA, United States; ^2^Brain Research Institute, David Geffen School of Medicine, University of California, Los Angeles, Los Angeles, CA, United States; ^3^Department of Molecular and Medical Pharmacology, University of California, Los Angeles, Los Angeles, CA, United States

**Keywords:** decision-making, loss aversion, aging, cortical thickness, posterior cingulate, neuroimaging

## Abstract

Decision-making strategies shift during normal aging and can profoundly affect wellbeing. Although overweighing losses compared to gains, termed “loss aversion,” plays an important role in choice selection, the age trajectory of this effect and how it may be influenced by associated changes in brain structure remain unclear. We therefore investigated the relationship between age and loss aversion, and tested for its mediation by cortical thinning in brain regions that are susceptible to age-related declines and are implicated in loss aversion — the insular, orbitofrontal, and anterior and posterior cingulate cortices. Healthy participants (*n* = 106, 17–54 years) performed the Loss Aversion Task. A subgroup (*n* = 78) provided structural magnetic resonance imaging scans. Loss aversion followed a curvilinear trajectory, declining in young adulthood and increasing in middle-age, and thinning of the posterior cingulate cortex mediated this trajectory. The findings suggest that beyond a threshold in middle adulthood, atrophy of the posterior cingulate cortex influences loss aversion.

## Introduction

The proportion of the global population that is 65 years or older is increasing faster than those of other age groups; it is estimated that by 2050, one in four people in North America and Europe, and one in six people worldwide, will be over 65 (United Nations, 2019). As older adults face a myriad of choices that involve uncertainty and loss across multiple domains, changes in decision-making can substantially impact their quality of life (Samanez-Larkin, 2013; MacLeod et al., 2017). Accordingly, the impact of aging on decision-making is of substantial interest (Löckenhoff, 2018; Lighthall, 2020). Findings have been mixed, showing worsening in some respects, particularly in more deliberative domains, such as applying decision rules (Brown and Ridderinkhof, 2009). Yet, older adults can show more optimal decision-making than their younger counterparts, especially for choices that rely on life experience and acquired knowledge (Li et al., 2013).

Many everyday decisions present a potential for loss, which increases in salience with age (Ebner et al., 2006; Depping and Freund, 2011; Mata and Hertwig, 2011; Löckenhoff, 2018). When making a choice that balances the chance of gain against the risk of loss, people of all ages tend to be risk averse and to accept a gamble only if the magnitude of the win vastly outweighs that of the loss. This phenomenon has been explained by loss aversion, which reflects the overweighing of losses compared to equivalent gains (Kahneman and Tversky, 1979; Tversky and Kahneman, 1992). Despite reports of greater loss aversion in adults over compared to under 40 (Arora and Kumari, 2015; Kurnianingsih et al., 2015; O’Brien and Hess, 2020), other studies find no differences (Li et al., 2013; Rutledge et al., 2016; Pachur et al., 2017; Seaman et al., 2018). This discrepancy could be due to nonlinear effects of age on loss aversion, the exclusion of middle-aged participants in comparisons of older and younger groups (Li et al., 2013), or different methods of measuring loss aversion (Rutledge et al., 2016; Seaman et al., 2018).

Although aversions to risk and loss are presumably evolutionarily adaptive mechanisms (Robson, 1996; Chen et al., 2005; Zhang et al., 2014; Hintze et al., 2015), extreme sensitivity to potential loss can impair decision-making during laboratory tasks (Benjamin and Robbins, 2007; Cassotti et al., 2014) and real-world choices (Mishina et al., 2010; Herweg and Mierendorff, 2013; Schleich et al., 2019), and by people with psychiatric pathologies, such as affective disorders (Stamatis et al., 2020; Xu et al., 2020). Notably, a curvilinear relationship exists between age and both real-world financial choices (Agarwal et al., 2009) and laboratory risky decision-making (Read and Read, 2004; Tymula et al., 2013; Di Rosa et al., 2017), with better performance by middle-aged adults than their younger or older counterparts.

The goal of this study was to determine whether loss aversion followed a curvilinear relationship with age, and whether such a relationship is mediated by thickness of the insula, ventromedial prefrontal/orbitofrontal cortex (OFC), and/or anterior and posterior cingulate cortices, all of which are particularly vulnerable to age-related atrophy and are implicated in loss aversion (Tom et al., 2007; Canessa et al., 2013; Markett et al., 2016). Because risky decision-making (Tymula et al., 2013; Di Rosa et al., 2017) and associated cognitive functions (Verhaeghen and Salthouse, 1997; Brockmole and Logie, 2013; Hartshorne and Germine, 2015) follow curvilinear trajectories with age, we hypothesized that age and loss aversion would be related by a quadratic function, and that cortical thickness would influence this relationship. Considering reports that the cortical regions selected for study exhibit linear age-related thinning (Tamnes et al., 2009; Lemaitre et al., 2012; Storsve et al., 2014), we hypothesized that cortical thickness would influence loss aversion after a threshold of atrophy had been reached. Loss aversion was measured using the Loss Aversion Task, and structural MRI was performed on participants from young adulthood through middle age (17 to 54 years).

## Materials and Methods

### Participants

Data presented here are from healthy, right-handed volunteers between the ages of 17 and 54 who participated in studies that were approved by the University of California, Los Angeles Institutional Review Board. 130 participants (40 women) completed the Loss Aversion Task and 24 were excluded during analysis of the behavioral data (see procedures for exclusion under *Loss Aversion Task* below), leaving 106 for final analysis. MRI and behavioral data from these participants, other than performance on the Loss Aversion Task, have been published in other reports (Dean et al., 2011, 2015, 2018, 2020; Ghahremani et al., 2011, 2012; Morales et al., 2012, 2015a,2015b; Payer et al., 2012; Zorick et al., 2012; Kohno et al., 2014; Ballard et al., 2015a, b; Jones et al., 2016; Okita et al., 2016a,b,c, 2018; Moeller et al., 2018; London et al., 2020). Recruitment utilized online and print advertisements. After initial screening, participants received detailed information about each study and gave written informed consent before screening for eligibility by physical examination, medical history, and psychiatric evaluation. Drug use history and demographic information were collected using questionnaires. Participants were excluded for medical or neurological disorders or any current Axis I psychiatric disorder except Nicotine Dependence, determined by the Structured Clinical Interview for DSM-IV (First et al., 1998). After intake, participants returned on a different day to perform the Loss Aversion Task, which was administered using identical procedures for all studies. A subset of participants (*n* = 83) also completed structural magnetic resonance imaging (sMRI) on a different day. Data from 5 of those participants were excluded during preprocessing, leaving 78 for analysis. The average time between behavioral testing and the sMRI scan was 7 days. At intake and on each test day, participants were required to provide a urine sample that was negative for amphetamine, cocaine, methamphetamine, benzodiazepines, opioids, and cannabis. They were compensated in the form of cash, gift cards, or vouchers.

### Loss Aversion Task

**FIGURE 1:**
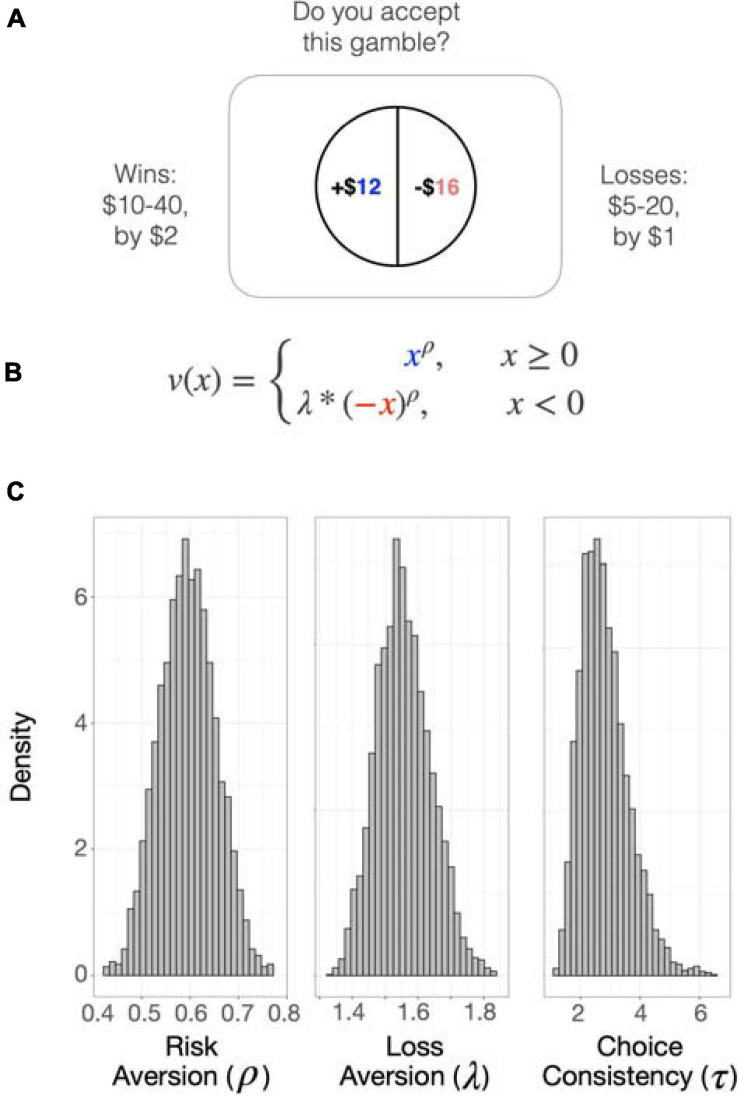
The Loss Aversion Task. **(A)** The task consisted of 128 sequential monetary choices to accept or reject a mixed gamble offering a 50/50 chance of winning a certain amount of money (blue) and losing a different amount of money (red). On each trial, an image representing a 50/50 choice was presented on the screen, and the participants indicated whether they strongly accept, weakly accept, weakly reject, or strongly reject the choice. Before testing, participants received thorough instruction and practice on how to perform the task. One choice was randomly selected to be paid out at the end of the task. **(B)**. Gain (*x*) and loss (*–x*) magnitudes of each choice were inserted into the subjective value equation *v(x)*. The loss aversion parameter (λ) represents the sensitivity to potential loss relative to potential gain. Rho (ρ) describes the curvature of the utility function and represents attitude toward risk. **(C)** Posterior distributions of parameters estimated using hierarchical Bayesian analysis, which enables the joint estimation of individual and group parameters. The distribution densities of each parameter are plotted. Higher values of λ indicate higher loss aversion and that the participant assigns more weight to losses than to gains of equal magnitude. When ρ < 1, the participant is risk-seeking for losses (more likely to take a gamble over a sure loss) and risk-averse for gains (more likely to choose a sure gain over a riskier prospect). The opposite is true when ρ > 1. Tau (τ) is the logit sensitivity and represents choice consistency, or the sensitivity of the participant to the difference between the certain amount and the gamble.

The task consisted of 128 sequential monetary choices to accept or reject a mixed gamble offering a 50/50 chance of winning a certain amount of money and losing a different amount of money (e.g., gaining $30 or losing $7) (Tom et al., 2007). On each trial, an image representing a 50/50 choice was presented on the screen, and the participants indicated whether they strongly accept, weakly accept, weakly reject, or strongly reject the choice (Figure 1A). Four options were provided instead of two (i.e., accept or reject) to discourage reliance on rule-based choice (e.g., always accepting when the loss exceeded $5). The probability of winning or losing was kept constant at 50%, and the alternative to accepting the gamble was always to remain at the status quo (i.e., win and lose nothing). The gains ranged from $10--40 in increments of $2, and the losses ranged from $5--20 in increments of $1. Once the participant decided, the next choice was presented without showing the outcome of the previous choice; if no selection was made within 3 s, the next gamble appeared on the screen. The task was presented using MATLAB (Mathworks, Natick, MA, United States) and the Psychtoolbox^1^ on an Apple PowerMac laptop computer running Mac OSX (Apple Computers, Cupertino, CA, United States), with most of the code being the same as used previously (Tom et al., 2007). Participants responded using the 1, 2, 3, and 4 keys on the keyboard.

Before testing, participants received thorough instruction on how to perform the task. Instructions were read aloud, and the participant was encouraged to ask questions while viewing training slides and performing 5–10 practice trials. To ensure that participants were motivated on the task, they were told that one of their choices would be randomly selected to be paid out at the end of testing. They also were told that losses would be deducted from their earnings from participation in the study, but losses were not actually deducted.

The data were assessed for quality and cleaned in two ways: (1) trials with implausible reaction times (i.e., <200 ms) were excluded (0.0048% of trials); (2) data were excluded for any participant whose preferences were random, erratic, or inconsistent with trends predicted by our structural model (i.e., they were not more likely to accept the gamble for increasing magnitude of gain, decreasing magnitude of loss, or increasing expected value). Data from 24 participants were excluded.

### Behavioral Choice Modeling

Choice parameters were estimated using a multi-parameter utility function (Sokol-Hessner et al., 2009) that represents subjective value (SV) (Eq. 1) based on original prospect theory (Kahneman and Tversky, 1979; Tversky and Kahneman, 1992):

(1) $$\text{SV}\,\left(x\right)=\left\{ \begin{array}{c} x^{\rho },x\geq 0\\ \lambda *{\left(-x\right)}^{\rho },x<0 \end{array}\right.$$

The SV of the gamble is estimated using the objective magnitudes of gain (*x*) and loss (−*x*)given in each choice and the parameters of loss aversion (lambda; λ) and risk attitude (rho; ρ) (Figure 1B). The sensitivity to potential loss relative to potential gain is represented by λ. If λ = 1, the participant values gains and losses equally. When λ > 1, the participant is considered loss averse and assigns more weight to losses than to gains of equal magnitude. When λ < 1, the participant is considered gain-seeking, and overvalues gains compared to losses. Rho (ρ) describes the curvature of the utility function and represents attitude toward risk. If ρ = 1, the participant’s preferences can be modeled by a linear utility function, which signifies that each incremental increase in reward has equal utility. Values for ρ other than one indicate that the preferences of the participant can be described by a utility function that shows diminishing marginal utility. When ρ < 1, the participant is risk-seeking for losses (more likely to take a gamble over a sure loss) and risk-averse for gains (more likely to choose a sure gain over a riskier prospect). The opposite is true when ρ > 1. We did not explicitly measure risk attitudes in either the loss or gain domains.

The subjective values were then inserted into a logit (softmax) function (Eq. 2) that estimates the probability of accepting the gamble based on the difference in SVs between the lottery (50/50 choice or SV_gamble_) and the fixed amount ($0 or SV_certain_). The responses “strongly accept” and “weakly accept” were both treated as accepting the gamble, and both “strongly reject” and “weakly reject” were treated as rejecting the gamble. Tau (τ) is the logit sensitivity and represents choice consistency, or the sensitivity of the participant to the difference between the certain amount and the gamble.

(2) $$p\,\left(\mathrm{Accept}\,\mathrm{Gamble}\right)={\left[1+\text{exp}\,\left(-\tau *S\,V_{\text{gamble}}-S\,V_{\text{certain}}\right)\right]}^{-1}$$

Parameter values were estimated using hierarchical Bayesian analysis with the “hBayesDM” package in R (Ahn et al., 2017), which enables the joint estimation of individual and group parameters and robustly identifies individual differences in decision-making (Ahn et al., 2011). Posterior inference was performed with Markov Chain Monte Carlo (MCMC) sampling using Stan (Carpenter et al., 2017) and RStan^2^. Models were validated by using the posterior distribution to generate data and visually inspecting whether the generated data corresponded to the underlying distribution.

### Structural MRI

Structural T1-weighted magnetic resonance images of the brain were acquired from 83 participants using a Magnetization Prepared Rapid Gradient Echo (MPRAGE) sequence (see Table 1). Images were collected from 31 participants on Scanner 1: a 1.5-Tesla Siemens Sonata MRI scanner (Erlangen, Germany) with a standard quadrature head coil (TR = 1900 ms, TE = 4.38 ms, flip angle = 15°, FOV = 160 mm × 256 mm × 256 mm, 176 slices, resolution: 1 mm × 1 mm × 1 mm). Images from 33 participants were collected on Scanner 2: a 3-Tesla Trio TIM Siemens MRI scanner (Erlangen, Germany) using parameters of TR = 2530 ms, TE = 3.31 ms, flip angle = 7°, FOV = 176 mm × 256 mm × 256 mm, 176 slices, resolution: 1 mm × 1 mm × 1 mm. Data from the remaining 14 participants were acquired on Scanner 3: a different 3-Tesla Trio TIM Siemens scanner using the same parameters.

**TABLE 1:** Demographics of participants tested on different scanners.

**Variable**	**Scanner 1 (1.5 T; *n* = 31)**	**Scanner 2 (3 T; *n* = 33)**	**Scanner 3 (3 T; *n* = 14)**	**Omnibus statistics**
Age, years^a^	32.8 (1.14)	19.9 (0.193)	38.0 (2.76)	*F*(2,75) = 61.1, *p* < 0.001***
Biological sex female/male (*n*)	18/13	8/25	4/10	χ^2^(2) = 8.38, *p* = 0.015*
IQ estimate standard score^a^	105.5 (2.153)	110.9 (1.843)	108.4 (2.408)	*F*(2,62) = 1.635, *p* = 0.203
Mother’s education, years^a^	12.3 (0.656)	14.8 (0.690)	13.3 (1.06)	*F*(2,72) = 3.16, *p* = 0.0482*
Race/ethnicity (*n*)				χ^2^(8) = 28.8, *p* < 0.001***
White	9	27	9	
African American	6	1	0	
Hispanic/Latinx	13	2	3	
Asian/Pacific Islander	0	3	1	
Other	3	0	1	
Cigarette smoking, n	13	14	10	χ^2^(2) = 3.94, *p* = 0.139

^*a*^Unless otherwise indicated, values are means (SE). IQ estimate = Weschler Test of Adult Reading. *p < 0.05; ***p < 0.001.

### MRI Processing

Anatomical MRI images were processed using FreeSurfer 6.0.0^3^, which generates a three-dimensional model of the cortical surface and provides measurements of local cortical thickness (Dale et al., 1999). Mean thickness within 72 automatically defined cortical parcels for each hemisphere were extracted from this model (Fischl et al., 2004; Desikan et al., 2006). Data quality was evaluated using the Qoala-T supervised learning quality control tool (Klapwijk et al., 2019), which identified data from 5 participants for exclusion, leaving data from the remaining 78 for the final analyses. As scans were acquired on different scanners, the ComBat procedure was used to harmonize the data and remove variability due to scanner type. ComBat has been validated on cortical thickness data and has been shown to robustly correct for scanner differences (Fortin et al., 2018). To preserve the variability due to age, we specified age as a biological variable for the ComBat model.

### Statistical Analysis

Statistical analyses were performed using RStudio version 1.1.456. Analysis of variance (ANOVA) or correlation, as appropriate, was used to determine whether λ was significantly associated with biological sex, race/ethnicity, estimated IQ [using the Wechsler Test of Adult Reading (WTAR) (Wechsler, 2001)], years of education of the participant’s mother (as a proxy for socioeconomic status), or cigarette smoking status. As shown below, only race/ethnicity was associated with λ and was therefore included as a covariate in subsequent analyses.

A generalized linear model (GLM) was used to assess the effect of age on loss aversion. The parameter estimate (λ) from the behavioral choice model was used as the dependent variable in a GLM with the independent variable of age. Based on previous research demonstrating a curvilinear relationship between age and economic decision-making under risk (Tymula et al., 2013), a hierarchical regression analysis was used to test for a quadratic relationship between λ and age, with age^2^ added as an independent variable for the second step of the model. On an exploratory basis, the same associations were tested with the risk attitude parameter, ρ.

The average of the mean cortical thickness of both hemispheres, weighted by cortical volume, was calculated to determine whether λ was related to whole-brain cortical thickness. Based on prior research indicating brain regions important for loss aversion (Tom et al., 2007; Canessa et al., 2013; Markett et al., 2016) and cortical thinning of the cortex with age (Tamnes et al., 2009; Lemaitre et al., 2012; Storsve et al., 2014), a region of interest (ROI) analysis was performed, including the insula, OFC, anterior cingulate cortex (ACC), and posterior cingulate cortex (PCC). ROIs were created by calculating a weighted average of both hemispheres for each region. A weighted average was also used to combine the rostral and caudal ACC to create one ACC ROI, and the medial and lateral OFC to create one OFC ROI.

To assess the main effect of cortical thickness on λ, a GLM was used for each region with λ as the dependent variable and the linear and quadratic components of cortical thickness (cortical thickness and the square of cortical thickness) as independent variables. Estimated intracranial volume was included as a covariate. Results were corrected for multiple comparisons using the Holm-Bonferroni method.

For brain regions showing significant relationships of structure with λ, a mediation analysis was performed to test whether cortical thickness mediated the relationship between age and λ. Age-related cortical thinning was confirmed using a GLM with cortical thickness as the dependent variable, age as the independent variable, and biological sex, race/ethnicity, and estimated intracranial volume tested as covariates. Age^2^ was then added as an independent variable for the second step of the model to check for any nonlinear effects of age.

The mediation model tested whether cortical thickness mediated the effect of age on λ. Because of the quadratic relationship between age and λ, age^2^ was specified as the independent variable, with age and estimated total intracranial volume as covariates. To account for any nonlinearities, the square of cortical thickness was also included as a covariate. The mediation analysis used the “mediations” specification of the “mediation” package in R, which enables nonparametric causal mediation analysis (Imai et al., 2010, 2013). Indirect effects, given by the Average Causal Mediation Effects (ACME), were computed using Monte Carlo simulations, and the 95% confidence intervals were computed by determining the effects at the 2.5 and 97.5th percentiles.

### Data Availability

All loss aversion task and cortical thickness data discussed in this manuscript, as well as the code used for statistical analyses, are publicly available at Open Science Framework under project title ‘‘Age Influences Loss Aversion Through Effects on Posterior Cingulate Cortical Thickness’’^4^.

## Results

### Relationship Between Loss Aversion and Demographic Variables

Biological sex, estimated IQ, cigarette smoking status, and years of mother’s education had no significant effects on λ (*p*s > 0.05), and, therefore, were not included in subsequent analyses (results were consistent when measures of socioeconomic status, such as father’s education, were used instead of mother’s education). An ANOVA revealed differences in λ based on race/ethnicity [*F*(4,101) = 5.78, *p* < 0.01], with post-hoc t-tests illustrating that Caucasians had higher λ than all other groups (*p*s < 0.05), and Hispanic/Latinx had higher λ than African Americans (*p* < 0.05); all other pairwise comparisons were nonsignificant (*ps* > 0.05). Based on these findings, subsequent analyses used race/ethnicity as a covariate which was coded as 1 = Caucasian, 2 = Hispanic/Latinx, 3 = African American, and 4 = Other.

### Quadratic Relationship Between Loss Aversion and Age

**FIGURE 2:**
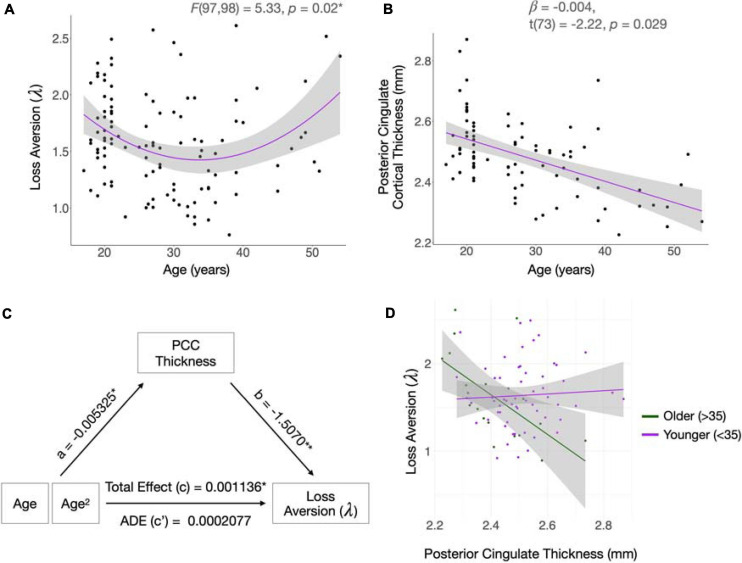
Relationships between age, loss aversion, and cortical thickness. **(A,B)** Loss aversion (λ) follows a quadratic trajectory with age, whereas cortical thickness of the posterior cingulate cortex (PCC) declines linearly with time. Shading indicates standard error confidence intervals. **(C)** Cortical thickness of the PCC mediates age-related changes in λ. The effect of age on PCC thickness is given by “a.” The effect of PCC thickness on λ is given by “b.” The Average Direct Effect (ADE; “c”) is the effect of age on λ when controlling for the mediator of PCC thickness. To calculate the Total Effect (c) of age on λ, without accounting for the mediator, both age and age^2^ were included in the model and the regression coefficient for age^2^ was taken as the strength of the effect. The causal mediation analysis was performed using nonparametric bootstrap confidence intervals and Monte Carlo simulations. The model included age, age^2^, race/ethnicity, scanner, and estimated intracranial volume, as well as PCC thickness as the mediator. Age^2^ was specified as the variable of interest. The measure of significance was given by the Average Causal Mediation Effect (ACME; *p* = 0.018*). Asterisks denote statistically significant results. *p < 0.05, **p < 0.01. **(D)** A negative relationship between PCC thickness and λ exists in older participants, but no relationship is present in participants under 35 years. The age of 35 was used to split the data into younger and older groups as it approximates the inflection point of the age-λ quadratic.

In data from the full sample, parameter estimates of the behavioral choice model, estimated using hierarchical Bayesian analysis, were consistent with published values (Tom et al., 2007; Sokol-Hessner et al., 2009, 2012). Posterior distributions of the parameters are shown in Figure 1C. Means with standard errors and ranges were: λ = 1.58 (0.04; 0.76 – 2.61; loss aversion), ρ = 0.60 (0.0036; 0.44 – 0.70; risk attitudes), τ = 3.07 (0.09; 0.96 – 6.74; choice consistency) and reaction time = 1.45 (0.0059; 0.206 – 4.49). When the quadratic variable of age was added to the model, both age [β = −0.067, *t*(97) = −2.24, *p* = 0.0*2*8] and age^2^ [β = 0.0010, *t*(97) = 2.309, *p* = 0.023] had significant effects, and the model fit the data better than the linear model [ANOVA; *F*(97,98) = 5.33, *p* = 0.02, change in *R*^2^ = 0.0433; Figure 2A].

The curvilinear association between λ and age persisted in the subsample from which sMRI data were acquired (*n* = 78); when the quadratic variable of age was added to the model, both age [β = −0.0722, *t*(75) = −2.36, *p* = 0.021] and age^2^ [β = 0.0011, *t*(75) = 2.46, *p* = 0.016] were significantly related to λ. The quadratic model provided a significantly better fit for the data than the linear model [ANOVA; *F*(75,76) = 6.074, *p* = 0.016; change in *R*^2^ = 0.070].

### Mediation by Posterior Cingulate Cortical Thickness of the Age Effect on Loss Aversion

#### Main Effects

Mean overall cortical thickness was not significantly related to loss aversion (β = 0.072, *t*(77) = 0.152, *p* = 0.88) and was therefore excluded from subsequent analyses. There were neither linear nor quadratic main effects of cortical thickness on λ in the insula [linear: β = −2.529, *t*(73) = −0.248, *p* = 0.805; quadratic: β = −0.467, *t*(73) = −0.278, *p* = 0.782], OFC [linear: β = 6.77, *t*(73) = 0.498, *p* = 0.620; quadratic: β = −1.32, *t*(73) = -0.515, *p* = 0.608], or ACC [linear: β = −1.209, *t*(73) = −1.071, *p* = 0.288; quadratic: β = 2.097, *t*(73) = 1.030, *p* = 0.306]. Although there were effects of both the linear and quadratic components of PCC thickness on λ [linear: β = −1.672, *t*(73) = −2.148, *p* = 0.035; quadratic: β = 3.30, *t*(73) = 2.13, *p* = 0.037], neither survived Holm-Bonferroni correction for multiple comparisons.

#### Mediation Analysis

Age-related cortical thinning of the PCC followed a linear course [β = −0.00728, *t*(73) = −5.19, *p* = 0.00000182; Figure 2B], with a small quadratic component [β = −0.000243, *t*(72) = 1.712, *p* = 0.091]. PCC thickness significantly mediated the age-loss aversion relationship, as quantified by the ACME (*p* = 0.028; Figure 2C). Since linear age-related change in the PCC was confirmed, but age and λ were quadratically related, we examined which component of the λ-age relationship was mediated by PCC thickness. To visualize the relationship between λ and PCC cortical thickness for different ages, we plotted the relationship between PCC thickness and λ by age for younger (<35) and older (>35) participants (Figure 2D). We split the data at the age of 35 as this was the inflection point of the age-loss aversion quadratic. The plot suggests that the mediation analysis captures an effect of PCC thickness on loss aversion that shifts throughout the lifespan, potentially mediating the increase in loss aversion in later life as opposed to the decrease in young adulthood.

### Exploratory Analyses: Risk Attitudes (ρ) and Brain Structure

The risk attitude parameter (ρ) was not significantly correlated with age [β = −0.000520, *t*(98) = 0.83, *p* = 0.408] or the quadratic variable of age [β = 0.0000266, *t*(97) = 0.494, *p* = 0.622]. There were no main effects for cortical thickness or the cortical thickness^2^ on risk attitudes in any of the four ROIs: insula [linear: β = 0.499, *t*(73) = 0.489, *p* = 0.626; quadratic: β = −0.0764, *t*(73) = −0.454, *p* = 0.651]; OFC [linear: β = −0.083, *t*(73) = −0.061, *p* = 0.951; quadratic: β = 0.0151, *t*(73) = 0.058, *p* = 0.954]; ACC [linear: β = −0.724, *t*(73) = −0.633, *p* = 0.529; quadratic: β = 0.130, *t*(73) = 0.631, *p* = 0.530]; PCC [linear: β = 1.144, *t*(73) = 1.439, *p* = 0.154; quadratic: β = −0.222, *t*(73) = −1.401, *p* = 0.165].

## Discussion

With the global population of those 65 years and older growing faster than all other age groups (United Nations, 2019), an understanding of the trajectory of decision-making over the lifespan may help people make better choices as they age (Agarwal et al., 2009; MacLeod et al., 2017). Providing unique insight into the relationship between aging and decision-making, this study found an association between age and loss aversion that followed a quadratic function, declining across young adulthood and reaching a minimum around age 35 before increasing in middle-age. We also showed that PCC thickness mediates the relationship between age and loss aversion, suggesting that cortical thinning of the PCC is likely one of several factors that contribute to changes in decision-making throughout the lifespan. Because we also confirmed that PCC thickness declines linearly with age (Tamnes et al., 2009; Lemaitre et al., 2012; Storsve et al., 2014), PCC thinning may emerge as an important factor in loss aversion when a certain threshold of atrophy begins in middle age.

A nonlinear relationship between age and loss aversion could unify seemingly conflicting results in the literature. Previous studies may have captured components of the quadratic relationship: participants aged 25–40 were less loss averse than those aged 41–55 (Arora and Kumari, 2015), and participants ∼18–28 were less loss averse than those aged ∼60–86 years (Kurnianingsih et al., 2015; O’Brien and Hess, 2020). Others may have missed differences due to the nonlinearities observed here (Li et al., 2013; Pachur et al., 2017). Our findings conflict with certain studies that did not find a quadratic relationship between age and loss aversion (Gächter et al., 2010; Rutledge et al., 2016; Seaman et al., 2018), which may be accounted for by the use of different tasks and methods to measure loss aversion (Gächter et al., 2010; Rutledge et al., 2016; Seaman et al., 2018). Nevertheless, the loss aversion and risk preference parameters were very similar to those recently reported in a study that fit a prospect theory utility function to choice data from 146 participants (Ackert et al., 2020).

The quadratic relationship between loss aversion and age mirrors the developmental trajectory of the cortex, during which the neurobiological mechanisms of cortical thinning differ in development and aging (Vidal-Pineiro et al., 2020). Cortical maturation includes thinning in sensory and eventually fronto-cortical areas, and may extend beyond the mid-twenties (Tamnes et al., 2009), whereas cortical thinning approaching middle-age could be considered the onset of senescence (Salat et al., 2004). Thus, PCC thickness may be unrelated to loss aversion during cortical maturation, but may arise as a contributing factor once cortical thinning is underway.

With normal aging, functional changes include the reduction of the integration of coordinated activity between brain regions and increases in the localization of function within regions (Bishop et al., 2010). Such reorganization can contribute to shifts in the mechanisms underlying decision-making, perhaps increasing reliance on certain regions and not others. The PCC has been linked to the representation of subjective value during probabilistic choice tasks (Kable and Glimcher, 2007; Levy et al., 2010), reward signaling (McCoy et al., 2003), attentional focus (Leech and Sharp, 2013), and the dynamic adaptation of behavior (Pearson et al., 2011). Beyond a threshold of cortical thinning of the PCC, such functions may be impeded, rendering the most adaptive strategy that which is the least cognitively demanding (Mata et al., 2007). Such adaptations could manifest in the use of an automatic or default heuristic, such as loss aversion, as shown by older adults using less cognitively taxing strategies in paradigms that involve risk (Weller et al., 2011). The plasticity of the brain coupled with an adaptive response to shifting cognitive resources (Gutchess, 2014) may result in older adults opting for choices that are “good enough” instead of searching to maximize outcomes [i.e., using “satisficing” instead of maximizing strategies (Kurnianingsih et al., 2015)]. During probabilistic choices involving loss, older adults are more likely to use such strategies when making decisions related to finances (Chen and Sun, 2003) and health (Besedeš et al., 2012). Satisficing strategies are related selectively to loss aversion and not to risk preferences; those who have greater loss aversion tend to stop searching for an optimal solution sooner (Schunk and Winter, 2009).

Notably, the Loss Aversion Task does not measure adaptive decision-making, and a loss-aversion strategy is not necessarily disadvantageous. Older individuals do not indiscriminately make worse decisions (Wood et al., 2005; Li et al., 2013, 2015; Bruine de Bruin et al., 2014), and heightened loss aversion may reflect naturally occurring shifts in values and motivations (Depping and Freund, 2011; Hess, 2014). Changes in cognitive faculties with age are not linear across time nor uniform across domains; the age-related decline of certain cognitive faculties, such as processing speed, episodic memory, and executive functions (Baltes and Lindenberger, 1997; Salthouse, 2019), may lead older adults to revert to a previously learned response, such as loss aversion, that requires less cognitive effort. Meanwhile, prioritizing the use of abilities that remain intact or even improve with age, such as those that depend on experience, emotional intelligence, and crystallized intelligence, may improve efficiency (Peters et al., 2007; Hess, 2014; Hartshorne and Germine, 2015; Zaval et al., 2015). Similarly, while young adults can take more risk than older adults, risk-seeking as measured in the laboratory is separable from loss aversion (Köbberling and Wakker, 2005). Thus, it is possible for a participant to display a certain level of loss aversion in the face of uncertain gambles but still be risk-seeking when presented different options.

The PCC also is implicated in emotional processing, as it is activated by emotional words (Maddock et al., 2003) and attending to emotional states (Terasawa et al., 2013). Emotional processing is necessary for adaptive decision-making (Loewenstein, 1996; Mellers et al., 1999; Phelps, 2009), and loss aversion is linked to the ability to regulate (Sokol-Hessner et al., 2009, 2012), and process (Bibby and Ferguson, 2011) emotions. Such faculties peak around age 45–60 (Hartshorne and Germine, 2015), and emotional content is particularly salient for older adults (Carstensen and Turk-Charles, 1994; Fung and Carstensen, 2003). Since reliance on emotional information can compensate for age-related declines in cognitively challenging situations (Hanoch et al., 2007; Peters et al., 2007), increases in loss aversion with age may reflect greater focus on emotional or experiential dimensions of decision-making. Related to emotional processing is interoception, which is also associated with the PCC (Kleckner et al., 2017; Stern et al., 2017) and tied to loss aversion (Sokol-Hessner et al., 2015). Thus, age-related cortical thinning in the PCC may hinder the ability to efficiently integrate affective responses into complex choices, especially those that include loss.

The present moment also gains salience with age, and prioritizing immediate or emotional wellbeing may intensify as time horizons constrict (Carstensen, 2006; Löckenhoff, 2011). Converging evidence, including self-reported goal orientations and performance on a probabilistic gambling task (Ebner et al., 2006; Depping and Freund, 2011; Mata and Hertwig, 2011), indicates a shift later in life toward avoiding losses instead of seeking gains. In fact, loss orientation in later adulthood is correlated with subjective well-being (Ebner et al., 2006). When motivations shift toward optimizing immediate, emotional wellbeing and processing power becomes limited with age, perhaps partly because cortical thinning of the PCC impedes probabilistic assessments, loss aversion may naturally emerge as a low-effort response when facing choices with uncertainty.

Higher loss aversion in younger participants and its subsequent decline across young adulthood may similarly reflect the underdevelopment of complex probabilistic decision-making (Weller et al., 2011; Beitz et al., 2014). The Loss Aversion Task requires the time-limited integration of the magnitude and probability of both reward and loss to decide whether the chance of reward is worth the risk of loss; this estimation of subjective value is critical to adaptive choice behavior. Sensitivity to the difference in expected value between options follows an inverted U-shaped function, suggesting that the ability to distinguish appropriately between reward-based options may not fully develop until the mid-20s (Weller et al., 2011).

While the age range of 17 to 54 covered in the current study does not represent the entire lifespan, prior studies point to the trajectory of the quadratic relationship observed here. Loss aversion was a main driver of behavior in children as young as 5–8 years old (Steelandt et al., 2013), and adults older than those examined here (aged 61–86) exhibited greater loss aversion than young adults (Kurnianingsih et al., 2015; O’Brien and Hess, 2020), consistent with the upward trend we observed from ages 35–54. Another limitation of this study is imbalance and relatively small samples of men and women; therefore, conclusive statements about effects of biological sex on loss aversion were not possible. That race/ethnicity was a significant factor in loss aversion also merits further investigation. The lack of an effect of age on risk-taking may reflect the type of task used, as the Loss Aversion Task is not necessarily designed to comprehensively elicit risk preferences. Finally, although there was no significant association between loss aversion and PCC thickness when correcting for multiple comparisons, lack of significance apparently reflected nonlinearities in the relationship – a negative correlation of loss aversion with PCC thickness in older participants, who had smaller PCC thickness, but not in participants whose PCC thickness crossed the inflection point on the U-shaped curve.

We conclude that cortical thickness of the PCC may supplement other cognitive and neurobiological age-related changes and arise as an important factor for loss aversion around the onset of age-related atrophy. Tracking age-related changes in the influence of decision-making biases, such as loss aversion, can inform policies that are tailored to the aging population (Samanez-Larkin, 2013). Moreover, determining the age at which changes begin can introduce opportunities for early intervention, such as services, education, or incentives that could better inform important life decisions, such as those related to health and finances (Johnson and Goldstein, 2003; Agarwal et al., 2009; MacLeod et al., 2017). Identification of brain regions that affect such choices when altered with age provides the opportunity to forecast – and perhaps forestall – future decision-making impairments. To this end, future longitudinal studies may go beyond cross-sectional investigations to use measurements from key brain regions (e.g., PCC) at mid-life to predict changes in decision making biases later in life.

## Data Availability Statement

The datasets presented in this study can be found in online repositories. The names of the repository/repositories and accession number(s) can be found below: Open Science Framework under project title “Age Influences Loss Aversion Through Effects on Posterior Cingulate Cortical Thickness” (https://osf.io/ejr56/).

## Ethics Statement

The studies involving human participants were reviewed and approved by the University of California, Los Angeles Institutional Review Board. The patients/participants provided their written informed consent to participate in this study.

## Author Contributions

DG, AD, EL, and ZG contributed to conception and design of the study. ZG performed the behavioral analysis and performed the statistical analyses and wrote the first draft of the manuscript. J-BP performed the cortical thickness analysis. All authors contributed to manuscript revision, and read and approved the submitted version.

## Conflict of Interest

The authors declare that the research was conducted in the absence of any commercial or financial relationships that could be construed as a potential conflict of interest.

## Footnotes

1
www.psychtoolbox.org

2 http://mc-stan.org/interfaces/rstan

3 http://surfer.nmr.mgh.harvard.edu

4 https://osf.io/ejr56/

## References

[B1] Ackert L. F. Deaves R. Miele J. Nguyen Q. (2020). Are time preference and risk preference associated with cognitive intelligence and emotional intelligence? *J. Behav. Financ.* 21 136–156. 10.1080/15427560.2019.1663850

[B2] Agarwal S. Driscoll J. C. Gabaix X. Laibson D. (2009). The age of reason: financial decisions over the life cycle and implications for regulation. *Brookings Pap. Econ. Act.* 2009 51–117. 10.1353/eca.0.0067

[B3] Ahn W. Y. Haines N. Zhang L. (2017). Revealing neurocomputational mechanisms of reinforcement learning and decision-making with the hBayesDM package. *Comput. Psychiatr.* 1 24–57. 10.1162/cpsy_a_00002 29601060 PMC5869013

[B4] Ahn W.-Y. Krawitz A. Kim W. Busmeyer J. R. Brown J. W. (2011). A model-based fMRI analysis with hierarchical bayesian parameter estimation. *J. neurosci. Psychol. Econ.* 4 95–110. 10.1037/a0020684 23795233 PMC3686299

[B5] Arora M. Kumari S. (2015). Risk taking in financial decisions as a function of age, gender: mediating role of loss aversion and regret. *Int. J. Appl. Psychol.* 5 83–89.

[B6] Ballard M. E. Dean A. C. Mandelkern M. A. London E. D. (2015a). Striatal dopamine D2/D3 receptor availability is associated with executive function in healthy controls but not methamphetamine users. *PLoS One* 10:e0143510. 10.1371/journal.pone.0143510 26657223 PMC4699455

[B7] Ballard M. E. Mandelkern M. A. Monterosso J. R. Hsu E. Robertson C. L. Ishibashi K. (2015b). Low dopamine D2/D3 receptor availability is associated with steep discounting of delayed rewards in methamphetamine dependence. *Int. J. Neuropsychopharmacol.* 18:yu119. 10.1093/ijnp/pyu119 PMC4540098 25603861

[B8] Baltes P. B. Lindenberger U. (1997). Emergence of a powerful connection between sensory and cognitive functions across the adult life span: a new window to the study of cognitive aging? *Psychol. Aging* 12 12–21. 10.1037/0882-7974.12.1.12 9100264

[B9] Beitz K. M. Salthouse T. A. Davis H. P. (2014). Performance on the Iowa gambling task: from 5 to 89 years of age. *J. Exp. Psychol. Gen.* 143 1677–1689. 10.1037/a0035823 24512562 PMC4115037

[B10] Benjamin A. M. Robbins S. J. (2007). The role of framing effects in performance on the balloon analogue risk task (BART). *Pers. Individ. Diff.* 43 221–230. 10.1016/j.paid.2006.11.026

[B11] Besedeš T. Deck C. Sarangi S. Shor M. (2012). Age effects and heuristics in decision making. *Rev. Econ. Stat.* 94 580–595. 10.1162/rest_a_00174 22544977 PMC3337688

[B12] Bibby P. A. Ferguson E. (2011). The ability to process emotional information predicts loss aversion. *Pers. Individ. Diff.* 51 263–266. 10.1016/j.paid.2010.05.001

[B13] Bishop N. A. Lu T. Yankner B. A. (2010). Neural mechanisms of ageing and cognitive decline. *Nature* 464 529–535. 10.1038/nature08983 20336135 PMC2927852

[B14] Brockmole J. R. Logie R. H. (2013). Age-related change in visual working memory: a study of 55,753 participants aged 8–75. *Front. Psychol.* 4:12. 10.3389/fpsyg.2013.00012 23372556 PMC3557412

[B15] Brown S. B. Ridderinkhof K. R. (2009). Aging and the neuroeconomics of decision making: a review. *Cogn. Affect. Behav. Neurosci.* 9 365–379. 10.3758/cabn.9.4.365 19897790

[B16] Bruine de Bruin W. Strough J. Parker A. M. (2014). Getting older isn’t all that bad: better decisions and coping when facing “sunk costs”. *Psychol. Aging* 29 642–647. 10.1037/a0036308 25244483 PMC4362707

[B17] Canessa N. Crespi C. Motterlini M. Baud-Bovy G. Chierchia G. Pantaleo G. (2013). The functional and structural neural basis of individual differences in loss aversion. *J. Neurosci.* 33 14307–14317. 10.1523/jneurosci.0497-13.2013 24005284 PMC6618376

[B18] Carpenter B. Gelman A. Hoffman M. D. Lee D. Goodrich B. Betancourt M. (2017). Stan: a probabilistic programming language. *J. Stat. Softw.* 76 1–32. 10.1017/9781108770750.002 PMC9788645 36568334

[B19] Carstensen L. L. (2006). The influence of a sense of time on human development. *Science* 312 1913–1915. 10.1126/science.1127488 16809530 PMC2790864

[B20] Carstensen L. L. Turk-Charles S. (1994). The salience of emotion across the adult life span. *Psychol. Aging* 9 259–264. 10.1037/0882-7974.9.2.259 8054174

[B21] Cassotti M. Aïte A. Osmont A. Houdé O. Borst G. (2014). What have we learned about the processes involved in the Iowa gambling task from developmental studies? *Front. Psychol.* 5:915. 10.3389/fpsyg.2014.00915 25191295 PMC4138612

[B22] Chen M. K. Lakshminarayanan V. Santos L. (2005). *The evolution of Our Preferences: Evidence from Capuchin Monkey Trading Behavior*. Cowles Foundation Discussion Papers 1524. New Haven, CT: Cowles Foundation for Research in Economics, Yale University.

[B23] Chen Y. Sun Y. (2003). Age differences in financial decision-making: using simple heuristics. *Educ. Gerontol.* 29 627–635. 10.1080/713844418

[B24] Dale A. M. Fischl B. Sereno M. I. (1999). Cortical surface-based analysis: I. Segmentation and surface reconstruction. *Neuroimage* 9 179–194. 9931268 10.1006/nimg.1998.0395

[B25] Dean A. C. Kohno M. Morales A. M. Ghahremani D. G. London E. D. (2015). Denial in methamphetamine users: associations with cognition and functional connectivity in brain. *Drug Alcohol Depend.* 151 84–91. 10.1016/j.drugalcdep.2015.03.004 25840750 PMC4447566

[B26] Dean A. C. Morales A. M. Hellemann G. London E. D. (2018). Cognitive deficit in methamphetamine users relative to childhood academic performance: link to cortical thickness. *Neuropsychopharmacology* 43 1745–1752. 10.1038/s41386-018-0065-1 29704001 PMC6006320

[B27] Dean A. C. Nurmi E. L. Morales A. M. Cho A. K. Seaman L. C. London E. D. (2020). CYP2D6 genotype may moderate measures of brain structure in methamphetamine users. *Addict. Biol.* 26:e12950. 32767519 10.1111/adb.12950 PMC7865018

[B28] Dean A. C. Sevak R. J. Monterosso J. R. Hellemann G. Sugar C. A. London E. D. (2011). Acute modafinil effects on attention and inhibitory control in methamphetamine-dependent humans. *J. Stud. Alcohol Drugs* 72 943–953. 22051208 10.15288/jsad.2011.72.943 PMC3211965

[B29] Depping M. K. Freund A. M. (2011). Normal aging and decision making: the role of motivation. *Hum. Dev.* 54 349–367. 10.1159/000334396

[B30] Desikan R. S. Ségonne F. Fischl B. Quinn B. T. Dickerson B. C. Blacker D. (2006). An automated labeling system for subdividing the human cerebral cortex on MRI scans into gyral based regions of interest. *Neuroimage* 31 968–980. 10.1016/j.neuroimage.2006.01.021 16530430

[B31] Di Rosa E. Mapelli D. Arcara G. Amodio P. Tamburin S. Schiff S. (2017). Aging and risky decision-making: new ERP evidence from the Iowa gambling task. *Neurosci. Lett.* 640 93–98. 10.1016/j.neulet.2017.01.021 28093302

[B32] Ebner N. C. Freund A. M. Baltes P. B. (2006). Developmental changes in personal goal orientation from young to late adulthood: from striving for gains to maintenance and prevention of losses. *Psychol. Aging* 21 664–678. 10.1037/0882-7974.21.4.664 17201488

[B33] First M. Spitzer R. Gibbon M. Williams J. (1998). *Structured Clinical Interview for DSM-IV Patient Edition (SCID-I/PV and SCID-I/NP Version 2.0).* New York, NY: Biometric Research Department.

[B34] Fischl B. Van Der Kouwe A. Destrieux C. Halgren E. Ségonne F. Salat D. H. (2004). Automatically parcellating the human cerebral cortex. *Cereb. Cortex* 14 11–22. 10.1093/cercor/bhg087 14654453

[B35] Fortin J.-P. Cullen N. Sheline Y. I. Taylor W. D. Aselcioglu I. Cook P. A. (2018). Harmonization of cortical thickness measurements across scanners and sites. *Neuroimage* 167 104–120. 10.1016/j.neuroimage.2017.11.024 29155184 PMC5845848

[B36] Fung H. H. Carstensen L. L. (2003). Sending memorable messages to the old: age differences in preferences and memory for advertisements. *J. Pers. Soc. Psychol.* 85 163–178. 10.1037/0022-3514.85.1.163 12872892

[B37] Gächter S. Johnson E. J. Herrmann A. (2010). *Individual-Level Loss Aversion in Riskless and Risky Choices”*. Discussion Papers 2010-20. Nottingham: The Centre for Decision Research and Experimental Economics, School of Economics, University of Nottingham.

[B38] Ghahremani D. G. Lee B. Robertson C. L. Tabibnia G. Morgan A. T. De Shetler N. (2012). Striatal dopamine D2/D3 receptors mediate response inhibition and related activity in frontostriatal neural circuitry in humans. *J. Neurosci.* 32 7316–7324. 10.1523/jneurosci.4284-11.2012 22623677 PMC3517177

[B39] Ghahremani D. G. Tabibnia G. Monterosso J. Hellemann G. Poldrack R. A. London E. D. (2011). Effect of modafinil on learning and task-related brain activity in methamphetamine-dependent and healthy individuals. *Neuropsychopharmacology* 36 950–959. 10.1038/npp.2010.233 21289606 PMC3077264

[B40] Gutchess A. (2014). Plasticity of the aging brain: new directions in cognitive neuroscience. *Science* 346 579–582. 10.1126/science.1254604 25359965

[B41] Hanoch Y. Wood S. Rice T. (2007). Bounded rationality, emotions and older adult decision making: not so fast and yet so frugal. *Hum. Dev.* 50 333–358. 10.1159/000109835

[B42] Hartshorne J. K. Germine L. T. (2015). When does cognitive functioning peak? The asynchronous rise and fall of different cognitive abilities across the life span. *Psychol. Sci.* 26 433–443. 10.1177/0956797614567339 25770099 PMC4441622

[B43] Herweg F. Mierendorff K. (2013). Uncertain demand, consumer loss aversion, and flat-rate tariffs. *J. Eur. Econ. Assoc.* 11 399–432. 10.1111/jeea.12004

[B44] Hess T. M. (2014). Selective engagement of cognitive resources: motivational influences on older adults’ cognitive functioning. *Perspect. Psychol. Sci.* 9 388–407. 10.1177/1745691614527465 26173272 PMC5911399

[B45] Hintze A. Olson R. S. Adami C. Hertwig R. (2015). Risk sensitivity as an evolutionary adaptation. *Sci. Rep.* 5:8242. 25649757 10.1038/srep08242 PMC4648446

[B46] Imai K. Keele L. Yamamoto T. (2010). Identification, inference and sensitivity analysis for causal mediation effects. *Stat. Sci.* 25 51–71.

[B47] Imai K. Tingley D. Yamamoto T. (2013). Experimental designs for identifying causal mechanisms. *J. R. Stat. Soc. Ser. A (Stat. Soc.)* 176 5–51. 10.1111/j.1467-985x.2012.01032.x

[B48] Johnson E. J. Goldstein D. (2003). Do defaults save lives? *Science* 302 1338–1339. 10.1126/science.1091721 14631022

[B49] Jones H. W. Dean A. C. Price K. A. London E. D. (2016). Increased self-reported impulsivity in methamphetamine users maintaining drug abstinence. *Am. J. Drug Alcohol Abuse* 42 500–506. 10.1080/00952990.2016.1192639 27398730 PMC5055455

[B50] Kable J. W. Glimcher P. W. (2007). The neural correlates of subjective value during intertemporal choice. *Nat. Neurosci.* 10 1625–1633. 10.1038/nn2007 17982449 PMC2845395

[B51] Kahneman D. Tversky A. (1979). Prospect theory: an analysis of decision under risk. *Econometrica* 47 263–292. 10.2307/1914185

[B52] Klapwijk E. T. Van De Kamp F. Van Der Meulen M. Peters S. Wierenga L. M. (2019). Qoala-T: a supervised-learning tool for quality control of FreeSurfer segmented MRI data. *Neuroimage* 189 116–129. 10.1016/j.neuroimage.2019.01.014 30633965

[B53] Kleckner I. R. Zhang J. Touroutoglou A. Chanes L. Xia C. Simmons W. K. (2017). Evidence for a large-scale brain system supporting allostasis and interoception in humans. *Nat. Hum. Behav.* 1:0069. 28983518 10.1038/s41562-017-0069 PMC5624222

[B54] Köbberling V. Wakker P. P. (2005). An index of loss aversion. *J. Econ. Theory* 122 119–131. 10.1093/acprof:oso/9780199972050.003.0006 33782627

[B55] Kohno M. Morales A. M. Ghahremani D. G. Hellemann G. London E. D. (2014). Risky decision making, prefrontal cortex, and mesocorticolimbic functional connectivity in methamphetamine dependence. *JAMA Psychiatry* 71 812–820. 10.1001/jamapsychiatry.2014.399 24850532 PMC4119006

[B56] Kurnianingsih Y. A. Sim S. K. Y. Chee M. W. L. Mullette-Gillman O. D. A. (2015). Aging and loss decision making: increased risk aversion and decreased use of maximizing information, with correlated rationality and value maximization. *Front. Hum. Neurosci.* 9:280. 10.3389/fnhum.2015.00280 26029092 PMC4429571

[B57] Leech R. Sharp D. J. (2013). The role of the posterior cingulate cortex in cognition and disease. *Brain* 137 12–32. 10.1093/brain/awt162 23869106 PMC3891440

[B58] Lemaitre H. Goldman A. L. Sambataro F. Verchinski B. A. Meyer-Lindenberg A. Weinberger D. R. (2012). Normal age-related brain morphometric changes: nonuniformity across cortical thickness, surface area and gray matter volume? *Neurobiol. Aging* 33 617.e611–617.e619. 10.1016/j.neurobiolaging.2010.07.013 PMC3026893 20739099

[B59] Levy I. Snell J. Nelson A. J. Rustichini A. Glimcher P. W. (2010). Neural representation of subjective value under risk and ambiguity. *J. Neurophysiol.* 103 1036–1047. 10.1152/jn.00853.2009 20032238

[B60] Li Y. Baldassi M. Johnson E. J. Weber E. U. (2013). Complementary cognitive capabilities, economic decision making, and aging. *Psychol. Aging* 28 595–613. 10.1037/a0034172 24040999 PMC4086863

[B61] Li Y. Gao J. Enkavi A. Z. Zaval L. Weber E. U. Johnson E. J. (2015). Sound credit scores and financial decisions despite cognitive aging. *Proc. Natl. Acad. Sci. U.S.A.* 112 65–69. 10.1073/pnas.1413570112 25535381 PMC4291658

[B62] Lighthall N. R. (2020). Neural mechanisms of decision-making in aging. *Wiley Interdiscip. Rev. Cogn. Sci.* 11:e1519. 31608583 10.1002/wcs.1519

[B63] Löckenhoff C. E. (2011). Age, time, and decision making: from processing speed to global time horizons. *Ann. N. Y. Acad. Sci.* 1235 44–56. 10.1111/j.1749-6632.2011.06209.x 22023567 PMC3757097

[B64] Löckenhoff C. E. (2018). Aging and decision-making: a conceptual framework for future research–a mini-review. *Gerontology* 64 140–148. 10.1159/000485247 29212070 PMC5828978

[B65] Loewenstein G. (1996). Out of control: visceral influences on behavior. *Organ. Behav. Hum. Decis. Process.* 65 272–292. 10.1006/obhd.1996.0028

[B66] London E. D. Okita K. Kinney K. R. Dean A. C. Mcclintick M. N. Rizor E. J. (2020). No significant elevation of translocator protein binding in the brains of recently abstinent methamphetamine users. *Drug Alcohol Depend.* 213:108104. 10.1016/j.drugalcdep.2020.108104 32570138 PMC9059651

[B67] MacLeod S. Musich S. Hawkins K. Armstrong D. G. (2017). The growing need for resources to help older adults manage their financial and healthcare choices. *BMC Geriatr.* 17:84. 10.1186/s12877-017-0477-5 28399818 PMC5387227

[B68] Maddock R. J. Garrett A. S. Buonocore M. H. (2003). Posterior cingulate cortex activation by emotional words: fMRI evidence from a valence decision task. *Hum. Brain Mapp.* 18 30–41. 10.1002/hbm.10075 12454910 PMC6871991

[B69] Markett S. Heeren G. Montag C. Weber B. Reuter M. (2016). Loss aversion is associated with bilateral insula volume. A voxel based morphometry study. *Neurosci. Lett.* 619 172–176. 10.1016/j.neulet.2016.03.029 27012426

[B70] Mata R. Hertwig R. (2011). How to model age-related motivational reorientations in risky choice. *Hum. Dev.* 54 368–375. 10.1159/000334943

[B71] Mata R. Schooler L. J. Rieskamp J. (2007). The aging decision maker: cognitive aging and the adaptive selection of decision strategies. *Psychol. Aging* 22 796–810. 10.1037/0882-7974.22.4.796 18179298

[B72] McCoy A. N. Crowley J. C. Haghighian G. Dean H. L. Platt M. L. (2003). Saccade reward signals in posterior cingulate cortex. *Neuron* 40 1031–1040. 10.1016/s0896-6273(03)00719-0 14659101

[B73] Mellers B. Schwartz A. Ritov I. (1999). Emotion-based choice. *J. Exp. Psychol. Gen.* 128 332–345.

[B74] Mishina Y. Dykes B. J. Block E. S. Pollock T. G. (2010). Why “good” firms do bad things: The effects of high aspirations, high expectations, and prominence on the incidence of corporate illegality. *Acad. Manag. J.* 53 701–722. 10.5465/amj.2010.52814578

[B75] Moeller S. J. Okita K. Robertson C. L. Ballard M. E. Konova A. B. Goldstein R. Z. (2018). Low striatal dopamine D2-type receptor availability is linked to simulated drug choice in methamphetamine users. *Neuropsychopharmacology* 43 751–760. 10.1038/npp.2017.138 28664927 PMC5809782

[B76] Morales A. M. Kohno M. Robertson C. L. Dean A. C. Mandelkern M. A. London E. D. (2015a). Gray-matter volume, midbrain dopamine D2/D3 receptors and drug craving in methamphetamine users. *Mol. Psychiatry* 20 764–771. 10.1038/mp.2015.47 25896164 PMC4440838

[B77] Morales A. M. Kohno M. Robertson C. L. Dean A. C. Mandelkern M. A. London E. D. (2015b). Midbrain dopamine D2/D3 receptor availability and drug craving are associated with mesocorticolimbic gray matter volume in methamphetamine users. *Mol. Psychiatry* 20 658–658. 10.1038/mp.2015.59 25993553

[B78] Morales A. M. Lee B. Hellemann G. O’neill J. London E. D. (2012). Gray-matter volume in methamphetamine dependence: cigarette smoking and changes with abstinence from methamphetamine. *Drug Alcohol Depend.* 125 230–238. 10.1016/j.drugalcdep.2012.02.017 22445480 PMC3427723

[B79] O’Brien E. L. Hess T. M. (2020). Differential focus on probability and losses between young and older adults in risky decision-making. *Aging Neuropsychol. Cogn.* 27 532–552. 10.1080/13825585.2019.1642442 31355695 PMC6987007

[B80] Okita K. Ghahremani D. G. Payer D. E. Robertson C. L. Dean A. C. Mandelkern M. A. (2016a). Emotion dysregulation and amygdala dopamine D2-type receptor availability in methamphetamine users. *Drug Alcohol Depend.* 161 163–170. 10.1016/j.drugalcdep.2016.01.029 26880595 PMC4792713

[B81] Okita K. Ghahremani D. G. Payer D. E. Robertson C. L. Mandelkern M. A. London E. D. (2016b). Relationship of alexithymia ratings to dopamine D2-type receptors in anterior cingulate and insula of healthy control subjects but not methamphetamine-dependent individuals. *Int. J. Neuropsychopharmacol.* 19:yv129. 10.1093/ijnp/pyv129 PMC4886668 26657175

[B82] Okita K. Mandelkern M. A. London E. D. (2016c). Cigarette use and striatal dopamine D2/3 receptors: possible role in the link between smoking and nicotine dependence. *Int. J. Neuropsychopharmacol.* 19:yw074. 10.1093/ijnp/pyw074 PMC5137283 27634830

[B83] Okita K. Morales A. M. Dean A. C. Johnson M. C. Lu V. Farahi J. (2018). Striatal dopamine D1-type receptor availability: no difference from control but association with cortical thickness in methamphetamine users. *Mol. Psychiatry* 23 1320–1327. 10.1038/mp.2017.172 28894300 PMC5847392

[B84] Pachur T. Mata R. Hertwig R. (2017). Who dares, who errs? Disentangling cognitive and motivational roots of age differences in decisions under risk. *Psychol. Sci.* 28 504–518. 10.1177/0956797616687729 28406375

[B85] Payer D. Nurmi E. Wilson S. Mccracken J. London E. D. (2012). Effects of methamphetamine abuse and serotonin transporter gene variants on aggression and emotion-processing neurocircuitry. *Transl. Psychiatry* 2:e80. 10.1038/tp.2011.73 22832817 PMC3309557

[B86] Pearson J. M. Heilbronner S. R. Barack D. L. Hayden B. Y. Platt M. L. (2011). Posterior cingulate cortex: adapting behavior to a changing world. *Trends Cogn. Sci.* 15 143–151. 10.1016/j.tics.2011.02.002 21420893 PMC3070780

[B87] Peters E. Hess T. M. Västfjäll D. Auman C. (2007). Adult age differences in dual information processes: implications for the role of affective and deliberative processes in older adults’ decision making. *Perspect. Psychol. Sci.* 2 1–23. 10.1111/j.1745-6916.2007.00025.x 26151915

[B88] Phelps E. A. (2009). “The study of emotion in neuroeconomics,” in *Neuroeconomics: Decision Making and the Brain*, eds Glimcher P. W. Camerer C. F. Fehr E. Poldrack R. A. (Amsterdam: Elsevier Academic Press), 233–250. 10.1016/b978-0-12-374176-9.00016-6

[B89] Read D. Read N. L. (2004). Time discounting over the lifespan. *Organ. Behav. Hum. Decis. Process.* 94 22–32. 10.1016/j.obhdp.2004.01.002

[B90] Robson A. J. (1996). A biological basis for expected and non-expected utility. *J. Econ. Theory* 68 397–424. 10.1006/jeth.1996.0023

[B91] Rutledge R. B. Smittenaar P. Zeidman P. Brown H. R. Adams R. A. Lindenberger U. (2016). Risk taking for potential reward decreases across the lifespan. *Curr. Biol. CB* 26 1634–1639. 10.1016/j.cub.2016.05.017 27265392 PMC4920952

[B92] Salat D. H. Buckner R. L. Snyder A. Z. Greve D. N. Desikan R. S. R. Busa E. (2004). Thinning of the cerebral cortex in aging. *Cereb. Cortex* 14 721–730. 10.1093/cercor/bhh032 15054051

[B93] Salthouse T. A. (2019). Trajectories of normal cognitive aging. *Psychol. Aging* 34 17–24. 10.1037/pag0000288 30211596 PMC6367038

[B94] Samanez-Larkin G. R. (2013). Financial decision making and the aging brain. *APS Obs.* 26 30–33. 23946614 PMC3740974

[B95] Schleich J. Gassmann X. Meissner T. Faure C. (2019). A large-scale test of the effects of time discounting, risk aversion, loss aversion, and present bias on household adoption of energy-efficient technologies. *Energy Econ.* 80 377–393. 10.1016/j.eneco.2018.12.018

[B96] Schunk D. Winter J. (2009). The relationship between risk attitudes and heuristics in search tasks: a laboratory experiment. *J. Econ. Behav. Organ.* 71 347–360. 10.1016/j.jebo.2008.12.010

[B97] Seaman K. L. Green M. A. Shu S. Samanez-Larkin G. R. (2018). Individual differences in loss aversion and preferences for skewed risks across adulthood. *Psychol. Aging* 33 654–659. 10.1037/pag0000261 29771547 PMC6002928

[B98] Sokol-Hessner P. Camerer C. F. Phelps E. A. (2012). Emotion regulation reduces loss aversion and decreases amygdala responses to losses. *Soc. Cogn. Affect. Neurosci.* 8 341–350. 10.1093/scan/nss002 22275168 PMC3594725

[B99] Sokol-Hessner P. Hartley C. A. Hamilton J. R. Phelps E. A. (2015). Interoceptive ability predicts aversion to losses. *Cogn. Emotion* 29 695–701. 10.1080/02699931.2014.925426 24916358 PMC4263686

[B100] Sokol-Hessner P. Hsu M. Curley N. G. Delgado M. R. Camerer C. F. Phelps E. A. (2009). Thinking like a trader selectively reduces individuals’ loss aversion. *Proc. Natl. Acad. Sci.* 106 5035–5040. 10.1073/pnas.0806761106 19289824 PMC2656558

[B101] Stamatis C. A. Puccetti N. A. Charpentier C. J. Heller A. S. Timpano K. R. (2020). Repetitive negative thinking following exposure to a natural stressor prospectively predicts altered stress responding and decision-making in the laboratory. *Behav. Res. Ther.* 129:103609. 10.1016/j.brat.2020.103609 32283350 PMC9881836

[B102] Steelandt S. Broihanne M.-H. Romain A. Thierry B. Dufour V. (2013). Decision-making under risk of loss in children. *PLoS One* 8:e52316. 10.1371/journal.pone.0052316 23349682 PMC3548654

[B103] Stern E. R. Grimaldi S. J. Muratore A. Murrough J. Leibu E. Fleysher L. (2017). Neural correlates of interoception: effects of interoceptive focus and relationship to dimensional measures of body awareness. *Hum. Brain Mapp.* 38 6068–6082. 10.1002/hbm.23811 28901713 PMC5757871

[B104] Storsve A. B. Fjell A. M. Tamnes C. K. Westlye L. T. Overbye K. Aasland H. W. (2014). Differential longitudinal changes in cortical thickness, surface area and volume across the adult life span: regions of accelerating and decelerating change. *J. Neurosci.* 34 8488–8498. 10.1523/jneurosci.0391-14.2014 24948804 PMC6608217

[B105] Tamnes C. K. Østby Y. Fjell A. M. Westlye L. T. Due-Tønnessen P. Walhovd K. B. (2009). Brain maturation in adolescence and young adulthood: regional age-related changes in cortical thickness and white matter volume and microstructure. *Cereb. Cortex* 20 534–548. 10.1093/cercor/bhp118 19520764

[B106] Terasawa Y. Fukushima H. Umeda S. (2013). How does interoceptive awareness interact with the subjective experience of emotion? An fMRI Study. *Hum. Brain Mapp.* 34 598–612. 22102377 10.1002/hbm.21458 PMC6870042

[B107] Tom S. M. Fox C. R. Trepel C. Poldrack R. A. (2007). The neural basis of loss aversion in decision-making under risk. *Science* 315 515–518. 10.1126/science.1134239 17255512

[B108] Tversky A. Kahneman D. (1992). Advances in prospect theory: cumulative representation of uncertainty. *J. Risk Uncertain.* 5 297–323. 10.1007/bf00122574

[B109] Tymula A. Rosenberg Belmaker L. A. Ruderman L. Glimcher P. W. Levy I. (2013). Like cognitive function, decision making across the life span shows profound age-related changes. *Proc. Natl. Acad. Sci. U.S.A.* 110 17143–17148. 10.1073/pnas.1309909110 24082105 PMC3801020

[B110] United Nations (2019). World Population Prospects 2019, Online Edition. Rev. 1. U.N. Department of Economic and Social Affairs–Population Division. New York, NY: United Nations.

[B111] Verhaeghen P. Salthouse T. A. (1997). Meta-analyses of age–cognition relations in adulthood: Estimates of linear and nonlinear age effects and structural models. *Psychol. Bull.* 122 231–249. 10.1037/0033-2909.122.3.231 9354147

[B112] Vidal-Pineiro D. Parker N. Shin J. French L. Grydeland H. Jackowski A. P. (2020). Cellular correlates of cortical thinning throughout the lifespan. *Sci. Rep.* 10:21803. 33311571 10.1038/s41598-020-78471-3 PMC7732849

[B113] Wechsler D. (2001). *Wechsler Test of Adult Reading: WTAR.* San Antonio, TX: Psychological Corporation.

[B114] Weller J. A. Levin I. P. Denburg N. L. (2011). Trajectory of risky decision making for potential gains and losses from ages 5 to 85. *J. Behav. Decis. Mak.* 24 331–344. 10.1002/bdm.690

[B115] Wood S. Busemeyer J. Koling A. Cox C. R. Davis H. (2005). Older adults as adaptive decision makers: evidence from the Iowa gambling task. *Psychol. Aging* 20 220–225. 10.1037/0882-7974.20.2.220 16029086

[B116] Xu P. Van Dam N. T. Van Tol M.-J. Shen X. Cui Z. Gu R. (2020). Amygdala–prefrontal connectivity modulates loss aversion bias in anxious individuals. *Neuroimage* 218:116957. 10.1016/j.neuroimage.2020.116957 32442639

[B117] Zaval L. Li Y. Johnson E. J. Weber E. U. (2015). “Chapter 8–Complementary contributions of fluid and crystallized intelligence to decision making across the life span,” in *Aging and Decision Making*, eds Hess T. M. Strough J. Löckenhoff C. E. (San Diego, CA: Academic Press), 149–168. 10.1016/b978-0-12-417148-0.00008-x

[B118] Zhang R. Brennan T. J. Lo A. W. (2014). The origin of risk aversion. *Proc. Natl. Acad. Sci. U.S.A.* 111 17777–17782. 25453072 10.1073/pnas.1406755111 PMC4273387

[B119] Zorick T. Lee B. Mandelkern M. A. Fong T. Robertson C. Ghahremani D. G. (2012). Low striatal dopamine receptor availability linked to caloric intake during abstinence from chronic methamphetamine abuse. *Mol. Psychiatry* 17 569–571. 10.1038/mp.2011.137 22024765 PMC4111106

